# Mantle cell lymphoma artificial intelligence prognostic index using hematoxylin and eosin histology

**DOI:** 10.1038/s41375-026-03036-8

**Published:** 2026-07-07

**Authors:** Jonas Lippl, Sarah Reinke, Karoline Koch, Ilske Oschlies, Stefan Schrod, Lukas Wolfseher, Paul Hüttl, Michael Huttner, Silvia Beà, Elias Campo, Marco Ladetto, Sergio Cogliatti, Mats Ehinger, Grzegorz Rymkiewicz, Valentina Tabanelli, Hilka Rauert-Wunderlich, Andreas Rosenwald, Rainer Spang, Eva Hoster, Martin Dreyling, Michael Altenbuchinger, Wolfram Klapper

**Affiliations:** 1 Department of Medical Bioinformatics, University Medical Center Göttingen, Göttingen, Germany; 2 Department of Pathology, Hematopathology Section, University Hospital Schleswig-Holstein, Campus Kiel, Kiel, Germany; 3 Department of Statistical genomics and systems genetics, European Molecular Biology Laboratory (EMBL), Heidelberg, Germany; 4 Department of Statistical Bioinformatics, University of Regensburg, Regensburg, Germany; 5 Institut d’Investigacions Biomèdiques August Pi i Sunyer (IDIBAPS), Barcelona, Spain; 6 Centro de Investigación Biomédica en Red de Cáncer (CIBERONC), Barcelona, Spain; 7 Facultat de Medicina i Ciències de la Salut, Universitat de Barcelona, Barcelona, Spain; 8 Hospital Clínic de Barcelona, Barcelona, Spain; 9 Department of Translational Medicine, Division of Hematology, University of Eastern Piedmont and SCDU Ematologia, Azienda Ospedaliera Santi Antonio e Biagio e Cesare Arrigo, Alessandria, Italy; 10 Institut of Pathology, Kantonsspital St.Gallen, St. Gallen, Switzerland; 11 Division of Pathology, Department of Clinical Sciences Lund, Lund University, Lund, Sweden; 12 Department of Cancer Pathomorphology, Maria Sklodowska-Curie National Research Institute of Oncology, Warsaw, Poland; 13 Divisione di Diagnosi Emolinfopatologica, Haematopathology Division, European Institute of Oncology, Milano, Italy; 14 Institute of Pathology, University of Würzburg, Würzburg, Germany; 15 Institute for Medical Information Processing, Biometry, and Epidemiology (IBE), Faculty of Medicine, LMU Munich, Munich, Germany; 16 Department of Medicine III, LMU University Hospital, Munich, Germany; 17 Department of Medicine III, Ludwig-Maximilians-University Hospital, Munich, Germany; 18 Hematology and Hematopathology Departments, Hospital Clínic Barcelona, Barcelona, Spain, August Pi i Sunyer Biomedical Research Institute (IDIBAPS), Barcelona, Spain; 19 Division of Hematology, Department of Translational Medicine, University of Eastern Piedmont, Novara, Italy “SS Antonio e Biagio e Cesare Arrigo” Hospital, Alessandria, Italy; 20 Hospital Clínic, IDIBAPS, Universitat de Barcelona, Barcelona, Spain; 21 Department of Computer Science, University of Torino, Torino, Italy; 22 Department of Immunotechnology, Lund University, Lund, Sweden; 23 Unit of Hematology, Department of Biotechnology and Health Sciences, University of Torino, Torino, Italy; Hematology Division 1U, “AOU Città della Salute e della Scienza di Torino”, Torino, Italy; 24 Department of Hematology, GELTAMO, Hospital Clínic of Barcelona, Barcelona, Spain; 25 Department of Hematology, Oncology and Pneumology, Comprehensive Cancer Center, University Medical School of the Johannes Gutenberg-University, Mainz, Germany; 26 Institute for Medical Information Processing, Biometry, and Epidemiology, Ludwig-Maximilians-Universität München, Munich, Germany; 27 Department of Oncology, Lund University Hospital, Lund, Sweden; 28 Department of Pathology, Haematopathology Section and Lymph Node Registry, University of Kiel/University Hospital Schleswig-Holstein, Kiel, Germany; 29 Charles University, First Faculty of Medicine, Institute of Pathological Physiology, Prague, Czech Republic; 30 Medical Department II, University Hospital Schleswig-Holstein, Kiel, Germany; 31 Institute of Pathology, University of Würzburg and Comprehensive Cancer Center(CCC) Mainfranken, Würzburg, Germany

**Keywords:** Health sciences, Risk factors

## Abstract

The clinical course of Mantle Cell Lymphoma (MCL) varies between individual patients. Early detection of risk is crucial to assign MCL patients to novel treatment strategies. Most of the established biomarkers of outcome require specifically trained pathologists or molecular analysis. Here we introduce MAIPI (MCL Artificial Intelligence Prognostic Index), a deep learning algorithm trained only on Hematoxylin and Eosin (H&E) images of diagnostic biopsies of *n* = 428 MCL patients from clinical trials to assess prognosis. The capability of MAIPI to predict disease outcome was validated in an independent cohort of *n* = 140 patients treated with immunochemotherapy with and without ibrutinib. MAIPI selects areas of interest by itself and provides prognostic information independent of the MCL International Prognostic Index (MIPI) and Ki67 and without the need of molecular testing or expert pathologists evaluation.

## Introduction

Mantle Cell Lymphoma (MCL) is a mature B-cell lymphoma accounting for only 3-10% of all lymphomas [1]. MCL is characterized by activating translocations involving *CCND1*, the gene encoding for cyclin D1, in the vast majority of cases [2]. The lymphoma predominantly present as nodal disease but extranodal sites are frequently involved too [3]. Moreover, a non-nodal leukemic variant of MCL (nnMCL) has been identified which mainly presents in spleen, blood, and bone marrow [4].

The outcome of MCL has improved over the last few decades [5]. However, a high-risk population of MCL does not benefit from the recent therapeutic improvement, and novel therapeutic approaches such as cell therapy are currently evaluated in this population [6]. High-risk MCL has so far mostly been studied retrospectively, but future therapeutic strategies require the identification of this subgroup of MCL at the time of diagnosis, since these patients may benefit from novel therapeutic strategies like cellular therapy applied early during the course of the disease [7].

The mantle cell lymphoma international prognostic score (MIPI) is widely accepted as an easy to assess tool for risk assessment of MCL based on age and clinical parameters, including performance status, lactate dehydrogenase (LDH), and leukocyte count [8]. Several parameters, such as cytology [9], cell proliferation by Ki67, gene expression and mutational profiles [10] add to the prognostic performance of the MIPI. Due to the current status of standardization [11] and the accessibility in pathology labs, cell proliferation by Ki67 staining, and *P53* aberrations by immunohistochemistry or molecular testing are the best-established factors adding to the prognostic power of MIPI [12–14]. However, assessment of cell proliferation requires training, strict adherence to guidelines, and assessment by experienced hematopathologists [9, 11]. Analysis of *TP53* mutations by next-generation sequencing (NGS) is a standardized technology in most diagnostic labs but takes several days to generate results and might not be easily assessable in low-income countries [15].

Since features of cytology and cell proliferation are morphologically assessable on hematoxylin and eosin (H&E) stained slides, deep learning-based image analyses provide a unique opportunity to improve risk-stratification and make the assessment fast, cost-effective, observer-independent [9], and widely accessible. However, data on MCL images are limited in the current literature [16].

We collected the largest dataset of MCL whole slide images (WSIs) or tissue micro arrays (TMA) stained for H&E to develop a risk scoring model. Using deep learning methods, we established the first image-based risk score for MCL (MCL Artificial Intelligence Prognostic Index, MAIPI) considering the two endpoints overall survival (OS) and time to progression (TTP). Combining MAIPI with the clinical risk score MIPI, revealed that the MAIPI adds statistically independent prognostic information to MIPI and improves the risk assessment of MCL patients. MAIPI’s underlying algorithms and models were selected to provide research access to the technology, encourage its use, and validate its performance according to prevailing legal norms.

## Material and methods

### Whole slide images and tissue micro array

**Fig. 1:**
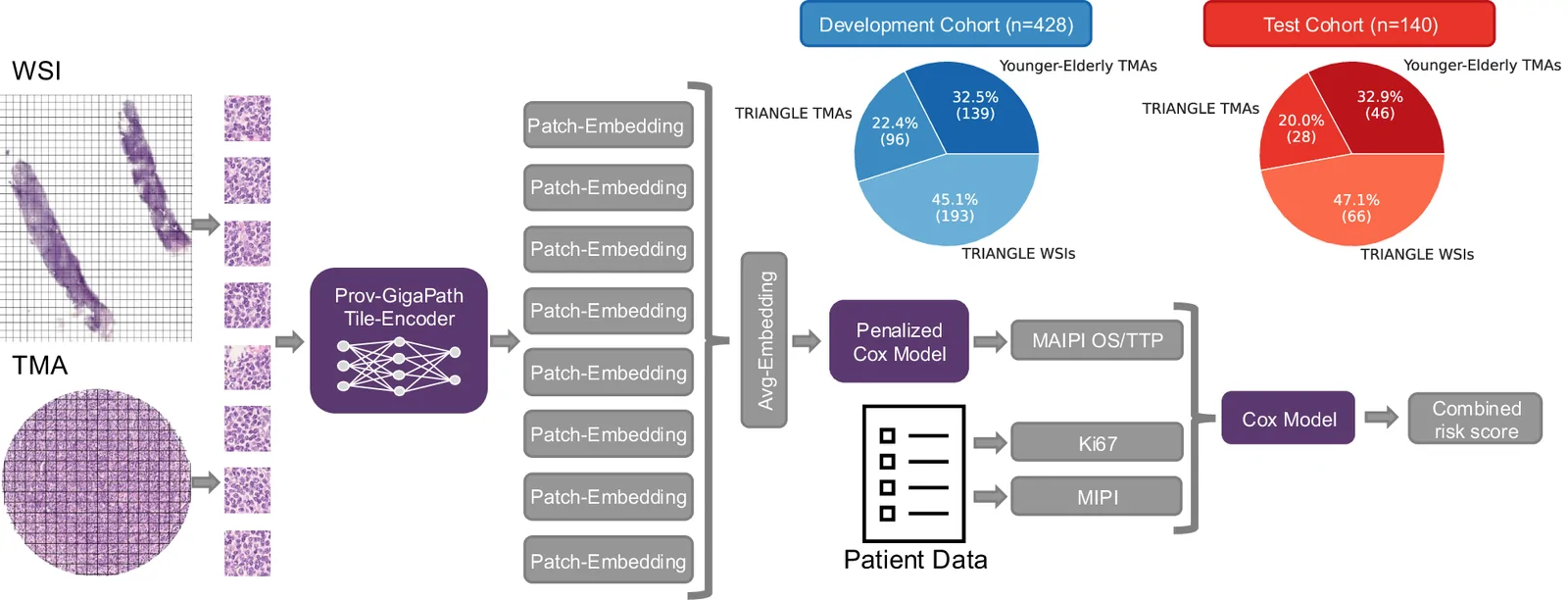
Visualization of the overall workflow. The TMAs and the WSIs are split into patches of the same size. From each WSI, the patches are ranked by their similarity to the average of TMA patches, allowing us to obtain a lightweight tumor classification and select eight representative patches. For the TMA patches and the selected WSI patches, 1532-dimensional embeddings are generated using the Prov-GigaPath model. On the embeddings of the development cohort, a penalized Cox model is trained in cross-validation to predict the MAIPI risk score for OS or TTP. The model is evaluated using the test cohort. Finally, the predicted scores can be combined with the existing clinical scores MIPI and the Ki67 biomarker to obtain a combined risk score.

The overall workflow of this study is illustrated in Fig. 1. TMAs stained with H&E were collected from patients in the MCL Younger [17] and MCL Elderly [18, 19] trials (*n* = 185), with trial registrations between 2004 and 2014. The follow-up period covered up to 16 years, with a median time-to-censoring of 9.35 years. In addition, whole slide images (WSI, *n* = 259) and TMA (*n* = 124) cases were gathered from the TRIANGLE [6] trial. For study protocols, register numbers, and other details of the study setting refer to the original trial publications [6, 17–19]. Since the Triangle cohort has a shorter follow-up (median time-to-censoring 4.66 years) and shows fewer events, patients from the two trials were randomly assigned into a development (*n* = 428) and test cohort (*n* = 140) (Fig. 1) (Baseline characteristics in Supplementary Table 1). For these, the MIPI (Supplementary Fig. 1A, B) or MIPIb (Supplementary Fig. 1C, D) (including Ki67) and expression of p53 were previously published [20] and assessed according to the published recommendations [9, 11]. Ki67 was available for *n* = 527 and p53 expression for *n* = 326 cases. Clinical end points were OS and TTP.

### Data pre-processing

Two H&E images for each patient reflecting the two tissue cores of each lymphoma in the TMA were generated at highest resolution (0.25 μm by 0.25 μm per pixel) using openslide [21] and assigned to their corresponding patient identity number. For cases with access to full slides, WSIs were generated. All images were converted into a SQLite database using the Pamly [22]. library for easy access.

Embeddings were generated using Prov-GigaPath [23] foundation model. This foundation model is available under the Apache 2.0 license, allowing us to share the MAIPI model with the research community according to prevailing legal norms. Prov-GigaPath is a vision transformer-based foundation model for computational pathology pre-trained in a self-supervised manner using DINOv2 on 171.189 WSIs to provide meaningful representations for patches. We sampled patches of size 224 × 224 px from WSIs and TMAs using Pamly [22] and each patch was mapped to a 1536-dimensional embedding using Prov-GigaPath.

The study leveraged two complementary data modalities, i.e., WSIs containing both tumor and non-tumor tissue, whereas TMAs are composed of pathologist-selected cores that are representative of tumor regions and optimally preserved for analysis. As a result, the entirety of the TMA cores is expected to reflect the complete morphological spectrum of MCL tissue that is well suited for biomarker assessment. This intrinsic difference between modalities introduces an imbalance between case and control samples, as WSIs contain substantial non-tumor content, posing challenges for model development. Based on these considerations, we calculated the cosine similarity between the average of all embeddings of the TMA patches and embeddings for each patch of the WSIs. We selected the eight patches from each WSI with the highest similarity scores to the entirety of TMA patches for further analysis (automated selection). Finally, the embeddings derived from the TMA cores or from the selected patches from the WSIs were averaged for further processing. One should note that these design choices are not unique. To establish an optimal processing strategy, we considered the cross-validated *C*-index on the training data to decide for the optimal number of representative patches and for the best aggregation strategy. For the number of representative patches, we explored 4,8,16, and 32 patches, and for the aggregation strategy, we tested aggregating the embeddings before model training and using multiple embeddings per patient and averaging the final predictions. The full pre-processing pipeline can be found at https://gitlab.gwdg.de/jlipl/cpathpipeline.

### Penalized Cox model development and validation

For model development, penalized Cox-Proportional Hazards (CoxPH) models with ElasticNet penalty [24] were fitted, where images represented by 1536-dimensional Prov-GigaPath embedding vector were used as predictor variables. In detail, a nested cross validation was performed on the development cohort for both hyper-parameter tuning and as a first, internal validation; first fivefold cross validation was performed to select the best set of hyper-parameters (penalty strength *α* and L1-ratio *r*). The set of hyper-parameters was subsequently used in 10 iterations of fivefold cross validation. This procedure yielded an ensemble of in total 50 models. Corresponding model predictions were collected and subsequently evaluated as internal validation. The model that was finally evaluated on the test data consists of the full ensemble of 50 models, where predictions are obtained by averaging across the models.

For performance evaluations, the concordance index (*C-*index) [25, 26] was used, where the 95% confidence interval (CI) was calculated via bootstrapping. For further visualization, we used Kaplan-Meier curves [27], where patients were stratified into four groups based on quartiles of the MAIPI, and into two groups based on a cut-point determined by minimizing the *P-*value on the validation predictions. This cut-point was subsequently applied for performance evaluations on the test cohort. We further established a CoxPH model that combines the MAIPI score from the H&E stained image with the MIPI score and, if available, the previously published Ki67 score. Respective model predictions were then compared via net reclassification improvement (NRI) [28] to the MIPI, MIPIb, and p53 expression values. Further, the **TRIPOD** + **AI Statement** [29] is available as supplementary material.

## Results

We collected H&E images of *n* = 568 MCL patients (*n* = 309 TMAs, *n* = 259 WSIs) treated in prospective randomized trials and generated the so far largest histology image collection of this rare entity. Our image analysis strategy allowed us to integrate WSIs and TMA cores in a single predictive model. Using embeddings generated by the pretrained foundational model (Prov-GigaPath) we applied a penalized CoxPH model for the learning task to predict OS or TTP. For the OS risk scoring, the best parameters of *α* = 0.12 and *r* = 0.15 were established and similarly *α* = 0.11 and *r* = 0.2 for the TTP task. The model’s result can be interpreted as a relative risk score MAIPI compared to the baseline hazard, which can be seen as the average hazard of an MCL patient within our cohort. Therefore, the score can range from 0 to approximately 10, although it has no theoretical upper limit.

**Fig. 2:**
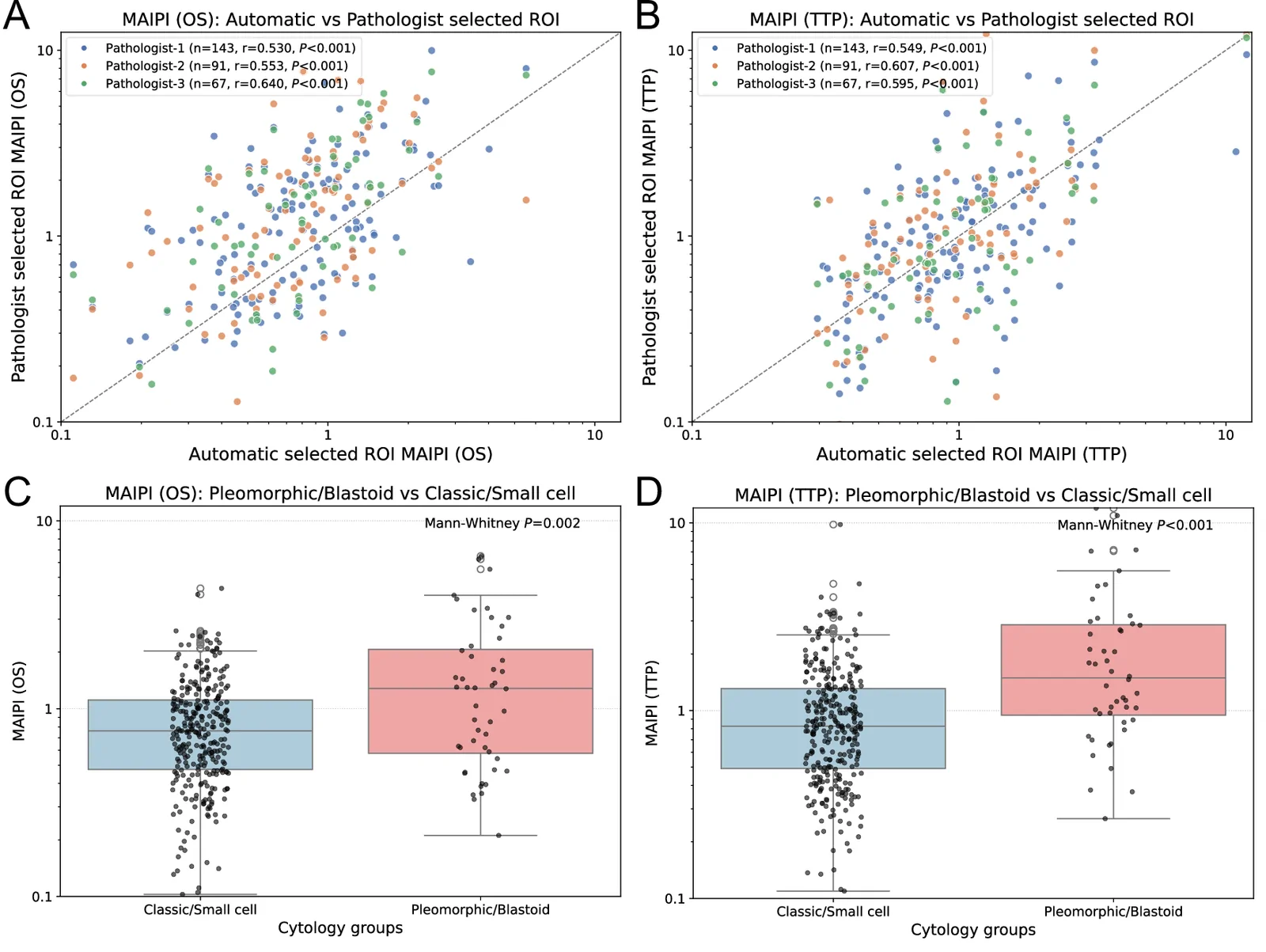
Robustness of MAIPI across ROI selection and cytology. Correlation between MAIPI for OS (**A**) and TTP (**B**) predicted for the average embeddings of eight automatically selected patches, and the MAIPI predicted for a single 2,048-pixel region of interest (ROI) selected by three different pathologists. As illustrated in the figure legend, the number of samples (n) from each pathologist, the Spearman correlation (r) and the corresponding *P*-value are given. Correlation between MAIPI and cytology variations of MCL for OS (**C**) and TTP (**D**).

First, we evaluated if the selection process of patches by MAIPI is reliable. For this purpose, we compared the MAIPI results of the AI-based automated patch-selection with regions of interest (ROI) selected by three expert pathologists independently as representative lymphoma tissue in the WSIs (range = 66–142 cases by each pathologist). The MAIPI for AI-based automated and expert pathologists ROI selection correlated with Spearman correlation coefficients between 0.530 and 0.640 (*P*-values < 0.001, Fig. 2A, B).

Having confirmed that our automated ROI selection reflects expert pathologist assessment, we studied the association of MAIPI with outcome. The MAIPI was fitted using five-fold cross-validation in the development cohort (*n* = 428), and each fit was repeated ten times with different seeds, establishing a total of 50 models. To evaluate the models, the scores on the test cohort (*n* = 140) were averaged, yielding *C*-indices of *C* = 0.673 *(P* = 0.003) for OS and *C* = 0.677 (*P* < 0.001) for TTP. The scores for each hold out validation fold were averaged after all iterations to provide independent scores for the full development cohort, yielding *C*-indices of *C* = 0.647 (*P* < 0.001) and *C* = 0.622 (*P* < 0.001) for OS and TTP (Supplementary Table 2).

**Fig. 3:**
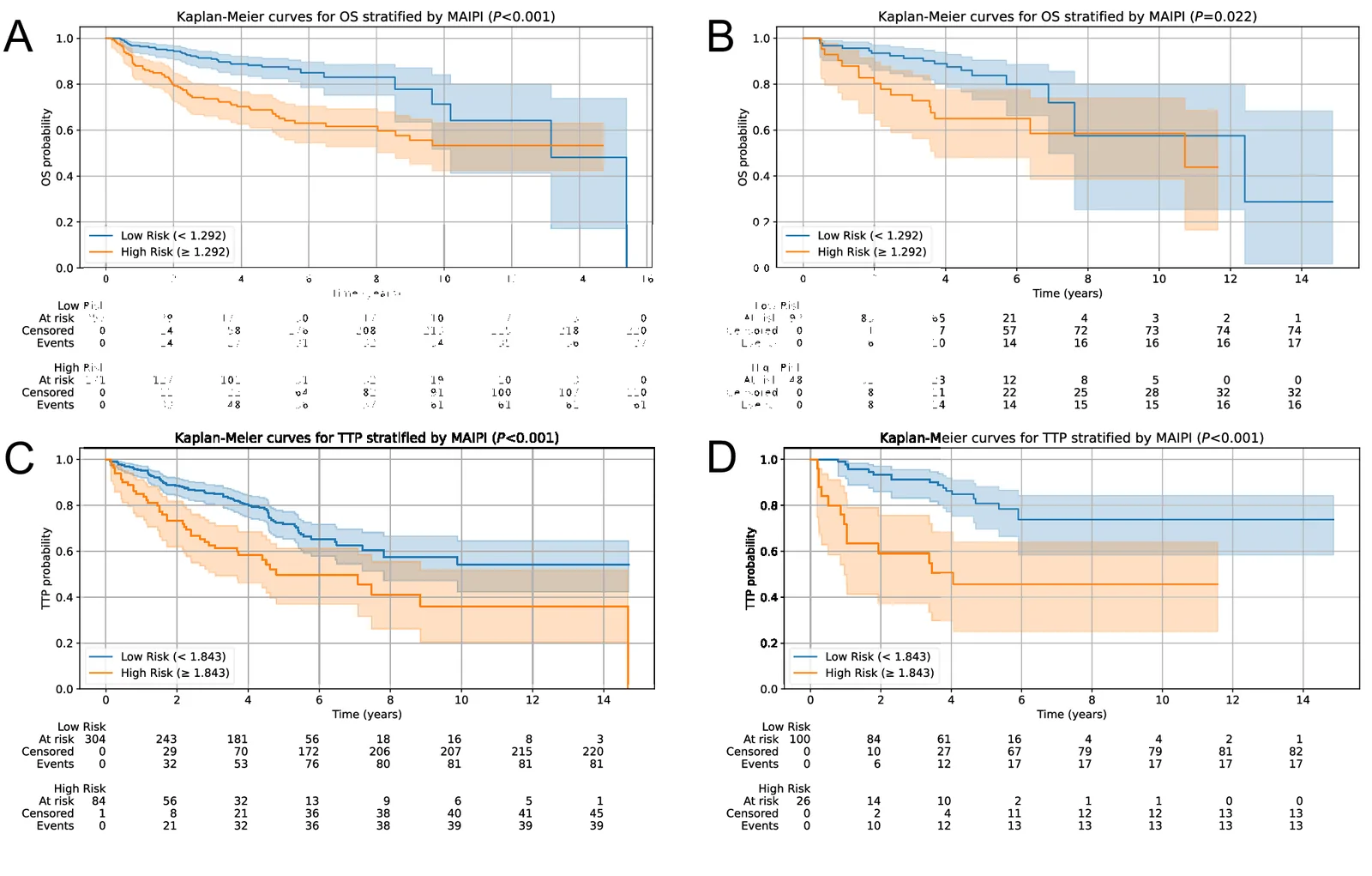
MAIPI stratifies OS and TTP outcomes. Kaplan-Meier curves for OS (**A**, **B**) and TTP (**C**, **D**) in the development cohort (**A**, **C**) and the independent test cohort (**B**, **D**). Patients were stratified by MAIPI based on an optimal threshold determined in the development cohort and subsequently applied to the test cohort.

Next, we tested for an association between the MAIPI and OS and TTP, respectively, using Cox-regression with MAIPI as a continuous predictor variable. For both OS and TTP a significant correlation was found with *P*-values < 0.001 (Supplementary Table 3). Using an unbiased stratification by quartiles, MAIPI was further significantly associated with OS and TTP (Supplementary Fig. 2) in the development and the test cohort (OS: *P* < 0.001 and *P* = 0.013, TTP: *P* = 0.018 and *P* = 0.013, respectively), indicating that MAIPI is a predictor of outcome as continuous variable. Prognostic information of MAIPI can be increased by optimized thresholds (1.292 for OS, 1.843 for TTP) selected on the development cohort and applied without any further fitting to the test cohort (OS: *P* < 0.001 and 0.022, TTP: *P* < 0.001 and *P* < 0.001, respectively, Fig. 3) to split patients into a high and a low risk group. To check for potential confounding variables, Cox-regression analysis was performed, including MIPI and Ki67 (Ki67 available for *n *= 527). The Cox-regression model was established using the validation predictions of the development cohort. When including only the MIPI score the MAIPI remained significant for OS with a *P-*value < 0.001 and <0.001 for TTP (Supplementary Table 4). This significance is also seen when calculating the *C*-index of this new combined risk score. We obtained for OS *C* = 0.692 (*P* < 0.001) on the development cohort and *C* = 0.657 (*P* = 0.004) on the test cohort, i.e. improvements of 0.027 and 0.002 compared to the MIPI score alone. This effect is even stronger for TTP, where we observed an improvement of 0.035 for the development cohort and 0.015 for the test cohort (Supplementary Table 2). When additionally including Ki67 in this analysis, MAIPI remains significant for OS (*P* = 0.023) and TTP (*P* = 0.039) (Supplementary Table 5). Combining all parameters (MAIPI, MIPI and Ki67) to a joint score showed only minor improvements in performance for OS and TTP, compared to MIPIb (Supplementary Table 2).

**Fig. 4:**
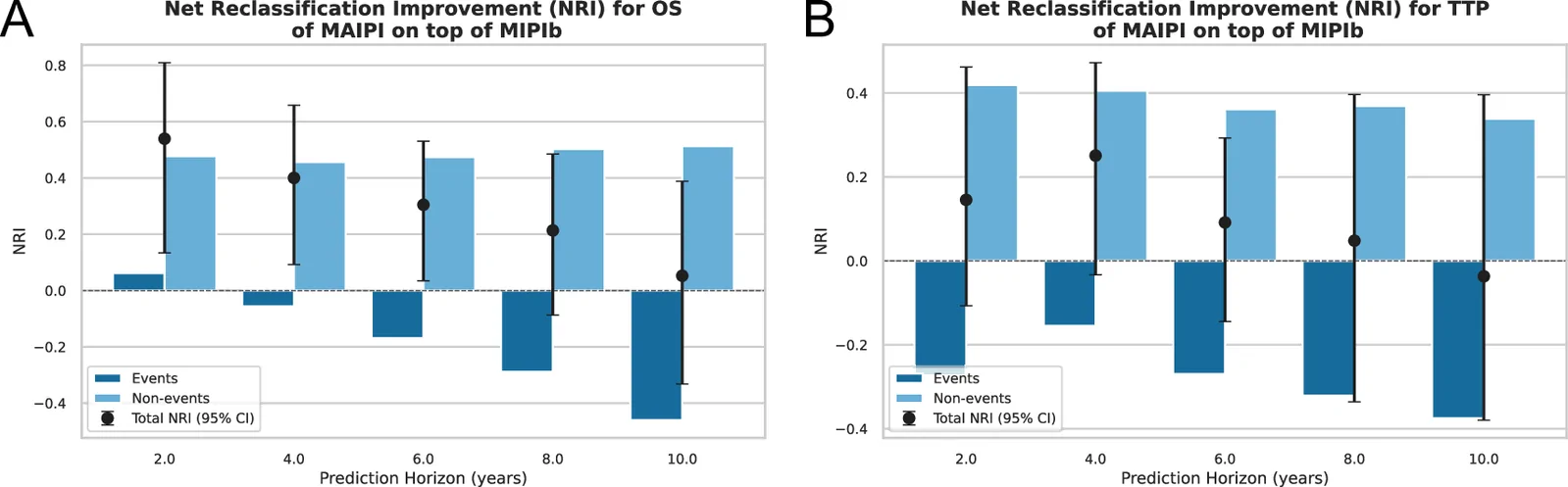
NRI analysis for MAIPI on top of MIPIb. Box plots for the results of the NRI analysis of performance added by MAIPI on top of the MIPIb at different time horizons. NRI was measured for events and non-events for the two outcomes OS (**A**) and TTP (**B**). The total NRI is the sum of the latter.

To further substantiate our findings, we established a joint model using MAIPI and MIPIb and compared it to MIPIb alone through an NRI analysis at different time horizons. We observed the most substantial reclassification improvements for a 2-year time horizon for OS (NRI 0.539, 95% CI 0.133–0.810, *P* = 0.024) and at 4 years for TTP (NRI 0.251, 95% CI –0.033 to –0.472, *P* = 0.084). In both analyses, the NRI was primarily driven by improved reclassification of patients without events; conversely, patients with events were only moderately better reclassified for OS at the 2-year horizon and slightly worse for TTP at the 4-year horizon (Fig. 4). We also noted that NRI values systematically decrease for longer time horizons in both cases.

To understand if MAIPI is associated with morphological features assessable by visual inspection, we further analyzed the distribution of MAIPI among cytological variants of MCL. As expected, MAIPI is higher in pleomorphic and blastoid MCL compared to classic or small cell variants (Fig. 2C, D, *P* = 0.001 for OS and *P* < 0.001 for TTP) [30]. Notably, we observed that the morphology of many lymphomas does not differ with respect to MAIPI (Fig. 2 and the illustrations in Supplementary Fig. 3). We hypothesize that MAIPI reflects complex or subtle morphological features that are undetectable by visual inspection. Interestingly, six lymphomas identified as the non-nodal variant of MCL using gene expression [31] showed a trend towards low MAIPI but the low number of non-nodal MCL included in this study prevented any definite conclusions (data not shown).

We further checked whether our score remained significant when we included p53 expression by immunohistochemistry in a Cox regression analysis (cut-off ≤50% versus >50%) [12]. For OS, MAIPI remains significant with a *P*-value of 0.026 but loses significance for TTP (Supplementary Table 6). Of note, this analysis was performed on a subset of cases (*n* = 244) of the development cohort due to the limited availability of p53 expression. Our previous findings align with our Kaplan-Meier analysis (Supplementary Fig. 1E, F), where we stratified patients within p53 ≤ 50% and p53 > 50% based on MAIPI for OS using the established threshold of 1.292. Stratification was significant for OS within the p53 > 50% group (*P* = 0.042) and slightly below the significance threshold (*P* = 0.054) within the p53 ≤ 50% group. For TTP, however, stratification by MAIPI was not significant for either group (data not shown). To check if our MAIPI provides clinical significance within the distinct treatment armsamong the patients treated in the most recent TRIANGLE trial, we calculated *C*-indices for each arm. For OS we obtained *C*-indices of *C* = 0.570 (*P* = 0.126), *C* = 0.661 (*P* = 0.065), and *C* = 0.611 (*P* = 0.073) for autologous transplantation, Ibrutinib, and a combination of both. For TTP, *C*-values of *C* = 0.622 (*P* = 0.008), *C* = 0.510 (*P* = 0.450), and *C* = 0.601 (*P* = 0.070) for autologous transplantation, Ibrutinib, and a combination of both were obtained.

## Discussion

The basis of risk stratification for MCL is the clinical risk score MIPI, which can be easily and reliably assessed by the treating physician. However, to stratify patients for treatment decisions, current concepts require the inclusion of additional tests such as Ki67 staining for cell proliferation [32, 33] and testing for *TP53* mutations [34]. These tests require experienced pathologists or molecular testing. The latter is time-consuming, costly, and probably unavailable in some parts of the world. Thus, relevant biomarker research in MCL should either improve risk-assessment or overcome limitations of existing biomarkers by being fast, cost-effective, and widely accessible. Here we introduce MAIPI and illustrate the power of deep learning to identify prognostic groups of lymphomas based on a cohort of H&E stained slides of MCL which is certainly unique in size and quality of clinical follow-up. Since MCL does not belong to the most frequent lymphomas, our image database may be unrivaled for some time and may thus serve as a reference for histology image analysis studies in this entity.

Compared to previously published methods of risk stratification of MCL, our image analysis tool is unique in several aspects. The time of analysis is only a few seconds since laboratory tests are not required. Moreover, the costs are minimal as the H&E stained slides are available anyhow to pathologists as part of the standard diagnostic procedure. Since we trained our algorithm on image sections, no scanning of the image is required, and pathologists can utilize a simple microscopic image file obtainable at any light microscope with a camera. Third and most importantly, this technology can be made accessible worldwide for research use.

Development of a tool unhampered by barriers was a major goal of our strategy to accelerate research in this relatively rare disease entity. The extent of access for the research community to challenge MAIPI in independent cohorts of MCL is critical. Whether other foundation models [35–38] bear the potential to increase the performance of MAIPI may be analyzed in future studies but was not the focus of our research. However, using at least one of these previously published foundation models (UNI [35]), we did not observe a major advantage of MAIPI compared to Prov-GigaPath [23] used in our analysis (data not shown). Thus, the potential benefits of other foundation models may be limited. To foster application by other research groups, MAIPI can now be accessed under: https://cau-git.rz.uni-kiel.de/FDLP/maipi. Multiple ways to implement MAIPI into clinical decision making can be envisioned, e.g., to be included in a combined score with both MIPI and Ki67 or to substitute Ki67 in combination with MIPI only. By replacing Ki67 with MAIPI, risk stratification becomes less vulnerable to staining, less costly, and less dependent on the pathologist’s individual experience. Moreover, MAIPI may be used to further dissect prognostic subgroups, e.g., the prognostic relevance of MAIPI seems strongest in the high Ki67 group of MCL (Supplementary Fig. 1G, H) and MAIPI may help to define a very high-risk group of MCL among the highly proliferative cases. Identification of such a high risk is currently the most critical medical need since this patient cohort did not sufficiently benefit from the recent advances in treatment [6]. In fact, since the MAIPI is potentially available at the time of diagnosis and highly significant for prognosis, it could serve as an alternative to the MIPI for making timely decisions.

Several open questions remain that warrant further studies. First, confirmation of the prognostic relevance of MAIPI in independent cohorts of MCL patients is certainly desirable. Second, we only studied MCL biopsies obtained at the time of first diagnosis. Whether MAIPI is able to predict outcome on relapse biopsies needs to be studied. In addition, combining MAIPI with in-depth molecular analysis in future studies will help us to understand which molecular features, other than cell proliferation, are associated with the image score. Third, the prognostic power of MAIPI in Ibrutinib-treated patients needs to be studied after longer follow-up and/or in independent cohorts. These future studies will also have to include attempts on how combining prognostic features like MIPI, MAIPI, Ki67, and TP53 mutational status will lead to an optimal risk stratification. Moreover, as larger datasets become available, more complex computational solutions, such as multiple instance learning methods, might become a viable alternative to the CoxPH model. These methods could be used to jointly learn relevant regions together with patient outcome. Finally, future studies may expand the application of AI beyond “class prediction” as in our study, e.g., for “class discovery” [39, 40]. We hope that the availability of MAIPI according to prevailing legal norms will foster such research and help to move this technology towards clinical decision-making.

## Supplementary information

Supplementary

Supplementary Figure 1

Supplementary Figure 2

Supplementary Figure 3

Supplementary Table 1

Supplementary Table 2

Supplementary Table 3

Supplementary Table 4

Supplementary Table 5

Supplementary Table 6

## Data Availability

Data is shared based on request and according to the ethic board recommendations for this study. Please contact the corresponding author.

## Code availability

The full training and analysis codes are available at https://cau-git.rz.uni-kiel.de/FDLP/maipi.

## Patient and public involvement

There was no patient or public involvement during design, conduct, reporting, interpretation, or dissemination of the study.
