# Supplementary material for: Mantle cell lymphoma artificial intelligence prognostic index using hematoxylin and eosin histology
Source: Leukemia. 2026 Jul 7;40(9):1961–7. doi: 10.1038/s41375-026-03036-8 (PMC13506322; doi:10.1038/s41375-026-03036-8)
Supplement: Supplementary file 1 [file 41375_2026_3036_MOESM2_ESM.pdf]

A

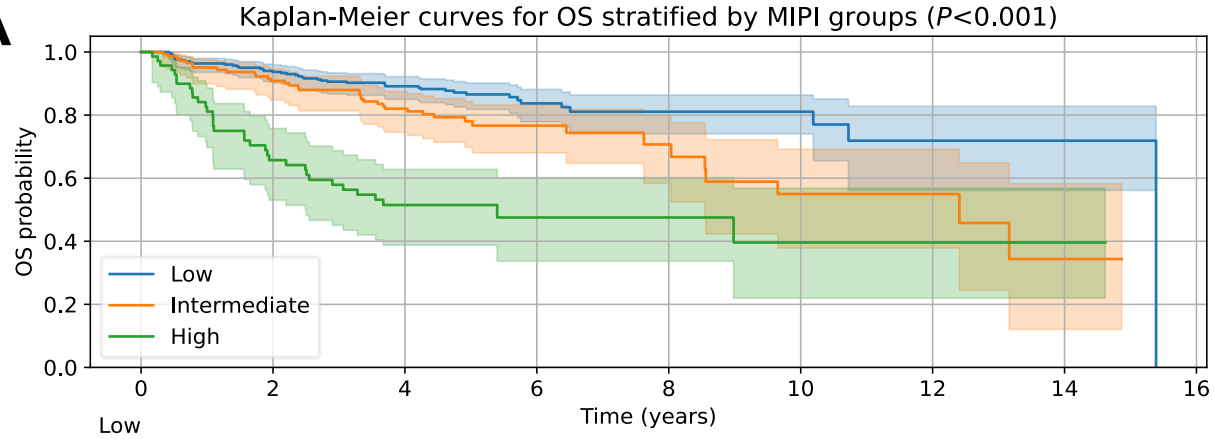

	0	2	4	6	8	10	12	14	16
**Low**									
At risk	310	276	216	77	31	20	11	4	0
Censored	0	15	62	193	237	248	255	262	265
Events	0	19	32	40	42	42	44	44	45
**Intermediate**									
At risk	147	128	99	38	18	13	6	2	0
Censored	0	6	23	79	97	98	105	107	109
Events	0	13	25	30	32	36	36	38	38
**High**									
At risk	70	42	27	8	6	4	2	1	0
Censored	0	5	11	29	31	32	34	35	36
Events	0	23	32	33	33	34	34	34	34

B

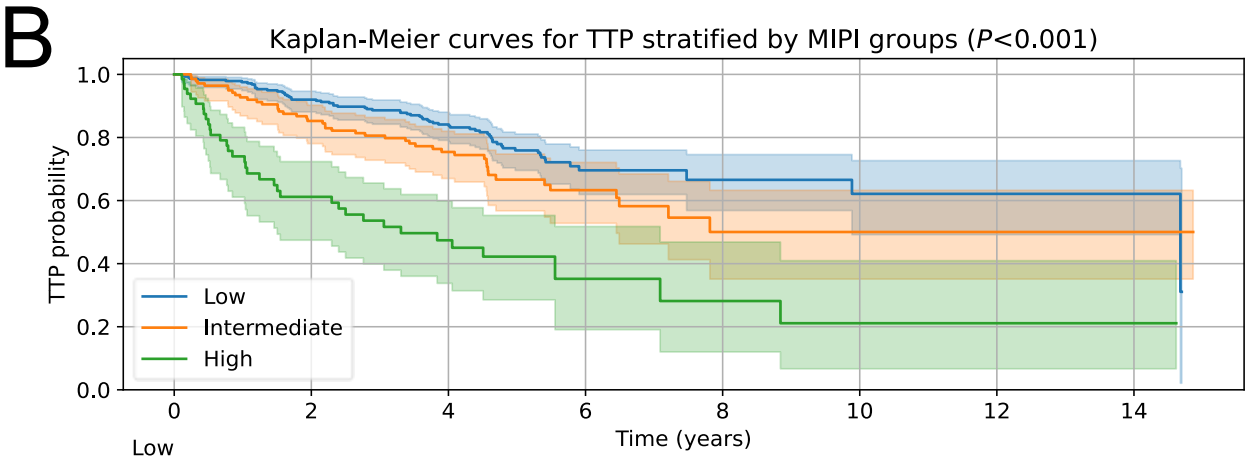

	0	2	4	6	8	10	12	14
**Low**								
At risk	295	249	182	53	17	14	9	3
Censored	0	24	71	179	214	216	221	227
Events	0	22	42	63	64	65	65	65
**Intermediate**								
At risk	143	112	80	28	11	10	4	1
Censored	0	11	31	74	87	88	94	97
Events	0	20	32	41	45	45	45	45
**High**								
At risk	68	33	20	5	4	3	2	1
Censored	0	12	18	30	30	30	31	32
Events	0	23	30	33	34	35	35	35

C

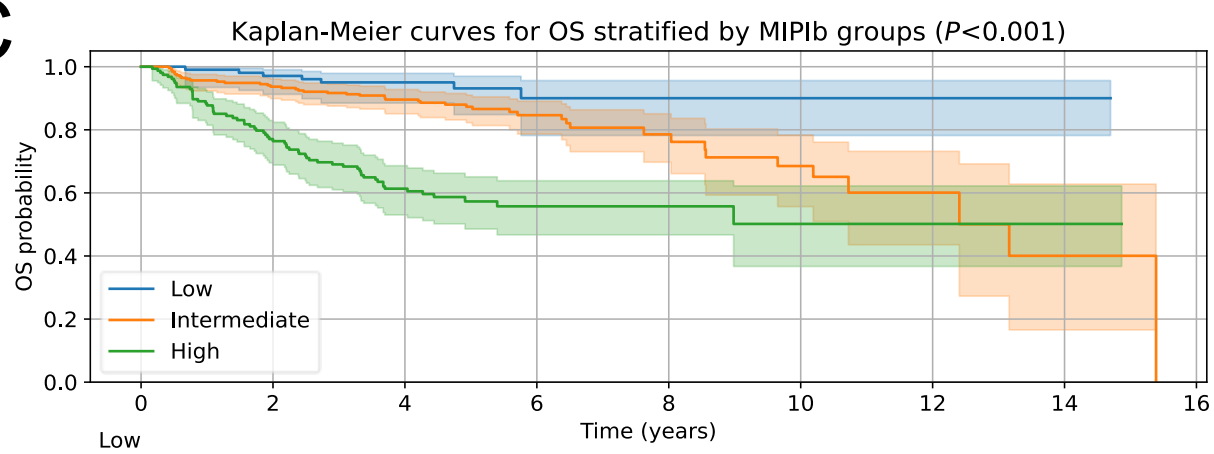

	0	2	4	6	8	10	12	14	16
**Low**									
At risk	107	97	73	27	12	8	7	2	0
Censored	0	7	29	73	88	92	93	98	100
Events	0	3	5	7	7	7	7	7	7
**Intermediate**									
At risk	264	235	192	75	33	21	6	2	0
Censored	0	13	46	156	194	202	215	217	218
Events	0	16	26	33	37	41	43	45	46
**High**									
At risk	156	114	77	21	10	8	6	3	0
Censored	0	6	21	72	83	84	86	89	92
Events	0	36	58	63	63	64	64	64	64

D

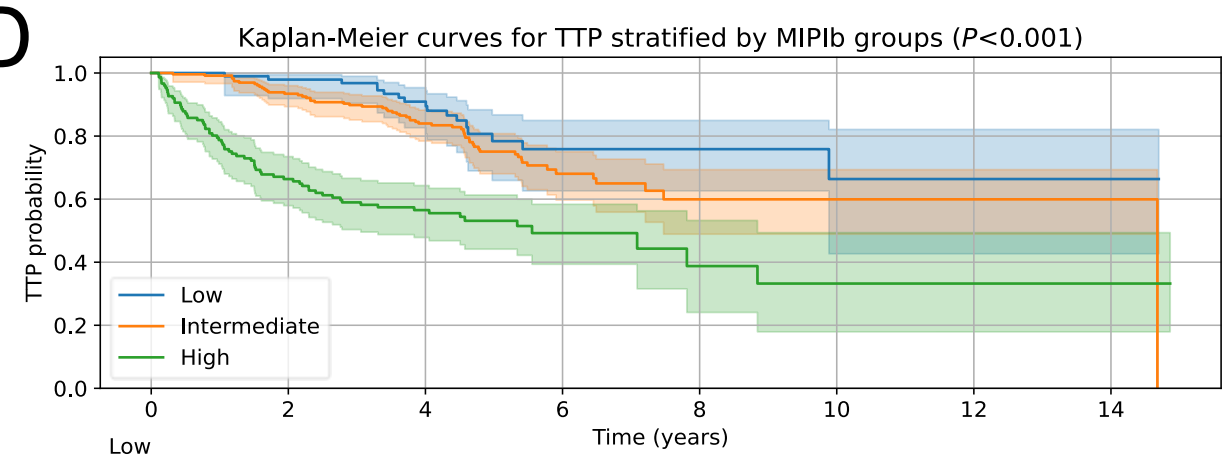

	0	2	4	6	8	10	12	14
**Low**								
At risk	102	93	63	19	8	7	6	2
Censored	0	7	31	67	78	78	79	83
Events	0	2	8	16	16	17	17	17
**Intermediate**								
At risk	250	210	157	50	17	14	5	1
Censored	0	25	58	145	174	177	186	190
Events	0	15	35	55	59	59	59	59
**High**								
At risk	154	91	62	17	7	6	4	2
Censored	0	15	31	71	79	79	81	83
Events	0	48	61	66	68	69	69	69

E

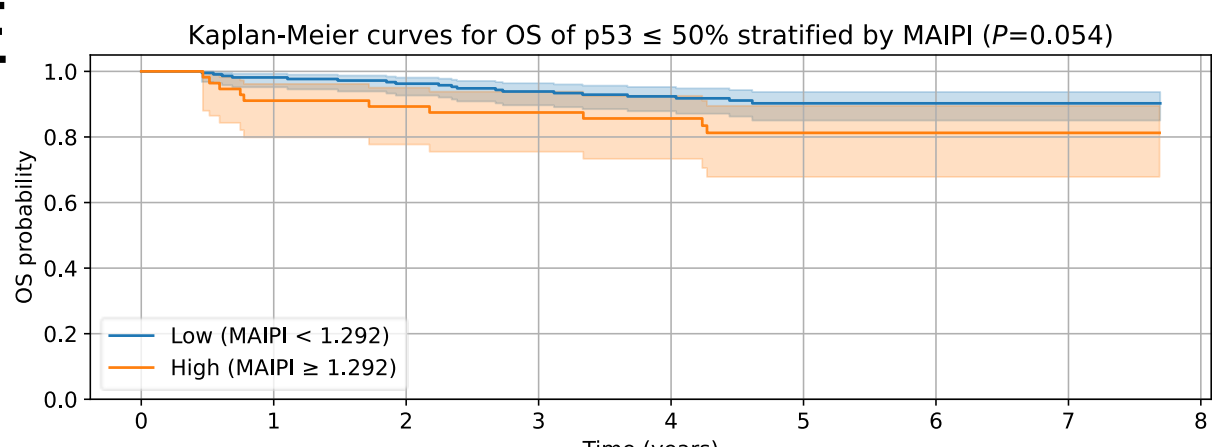

	0	1	2	3	4	5	6	7	8
**Low Risk**									
At risk	221	209	202	196	160	86	39	6	0
Censored	0	8	11	12	45	116	163	196	202
Events	0	4	8	13	16	19	19	19	19
**High Risk**									
At risk	56	51	49	48	45	21	10	4	0
Censored	0	0	1	1	3	25	36	42	46
Events	0	5	6	7	8	10	10	10	10

F

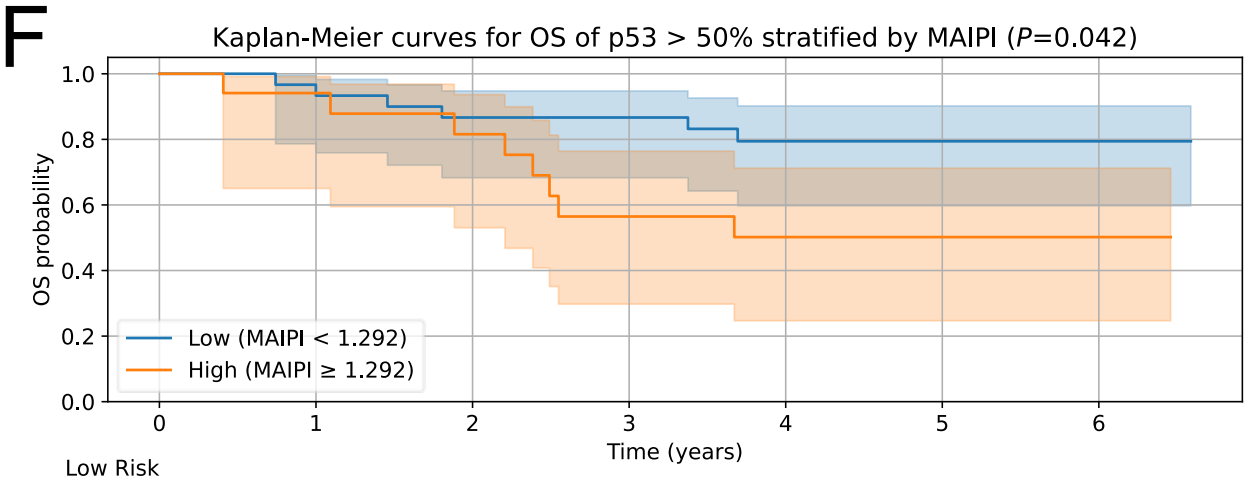

	0	1	2	3	4	5	6
**Low Risk**							
At risk	32	28	26	25	19	13	4
Censored	0	2	2	3	7	13	22
Events	0	2	4	4	6	6	6
**High Risk**							
At risk	17	15	13	9	6	4	1
Censored	0	1	1	1	3	5	8
Events	0	1	3	7	8	8	8

G

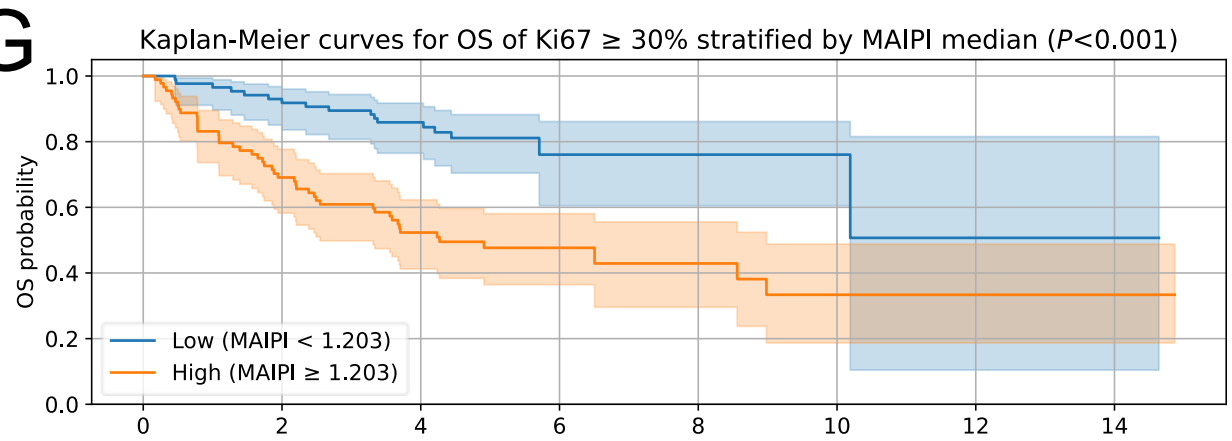

	0	2	4	6	8	10	12	14
**Low Risk**								
At risk	89	78	61	13	3	3	2	1
Censored	0	4	16	60	70	70	70	71
Events	0	7	12	16	16	16	17	17
**High Risk**								
At risk	90	59	39	15	9	6	6	2
Censored	0	4	10	31	36	37	37	41
Events	0	27	41	44	45	47	47	47

H

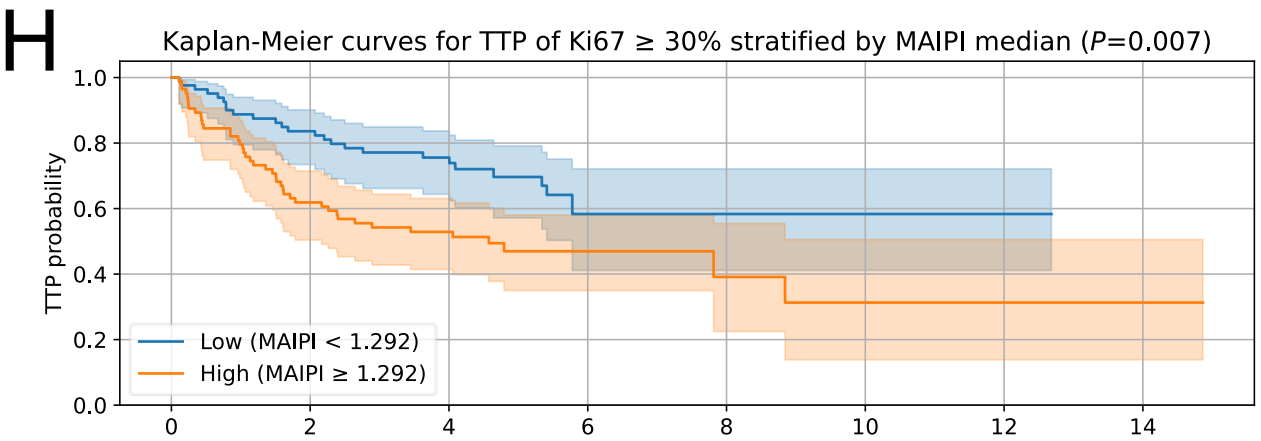

	0	2	4	6	8	10	12	14
**Low Risk**								
At risk	87	65	45	10	2	2	1	0
Censored	0	9	23	52	60	60	61	62
Events	0	13	19	25	25	25	25	25
**High Risk**								
At risk	88	49	36	9	5	4	4	1
Censored	0	8	14	38	41	41	41	44
Events	0	31	38	41	42	43	43	43
