# Supplementary material for: Mantle cell lymphoma artificial intelligence prognostic index using hematoxylin and eosin histology
Source: Leukemia. 2026 Jul 7;40(9):1961–7. doi: 10.1038/s41375-026-03036-8 (PMC13506322; doi:10.1038/s41375-026-03036-8)
Supplement: Supplementary file 2 [file 41375_2026_3036_MOESM3_ESM.pdf]

A

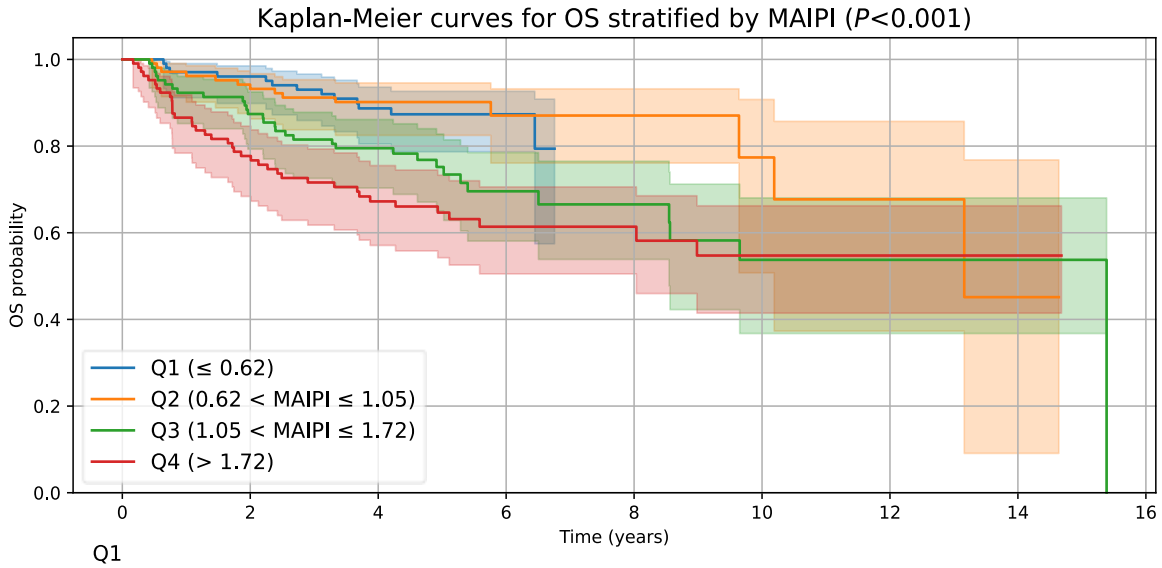

Q1									
At risk	107	96	70	15	0	0	0	0	0
Censored	0	7	26	80	94	94	94	94	94
Events	0	4	11	12	13	13	13	13	13
Q2									
At risk	107	94	76	28	13	8	6	2	0
Censored	0	6	21	68	83	87	88	91	93
Events	0	7	10	11	11	12	13	14	14
Q3									
At risk	107	89	68	26	16	10	4	2	0
Censored	0	5	18	54	63	66	72	74	75
Events	0	13	21	27	28	31	31	31	32
Q4									
At risk	107	77	59	32	20	11	7	2	0
Censored	0	7	15	38	50	57	61	66	68
Events	0	23	33	37	37	39	39	39	39

B

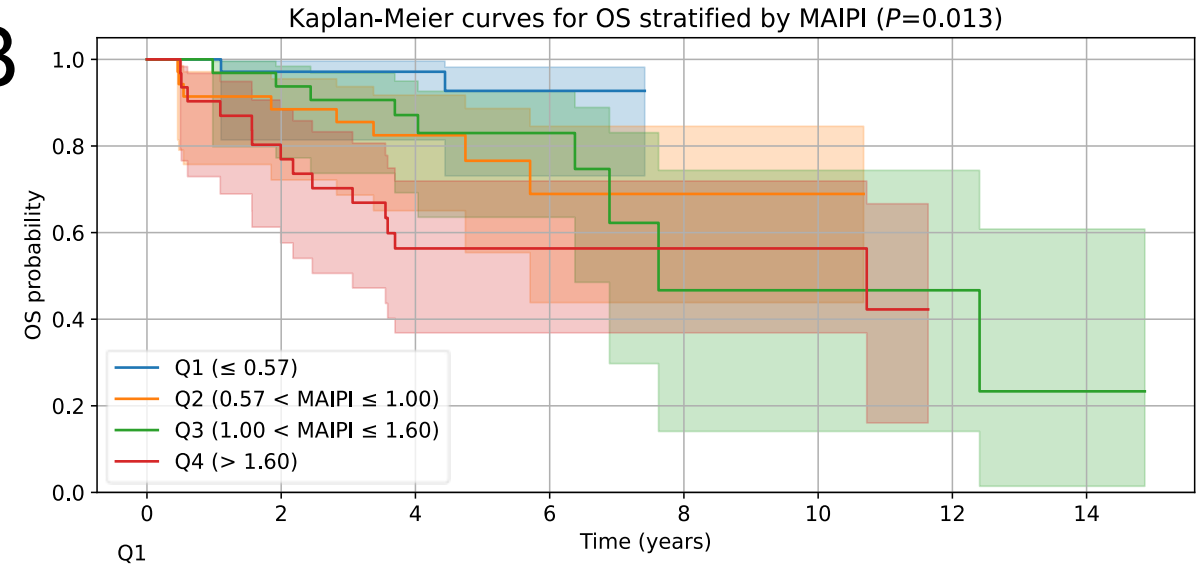

Q1								
At risk	35	34	25	4	0	0	0	0
Censored	0	0	9	29	33	33	33	33
Events	0	1	1	2	2	2	2	2
Q2								
At risk	35	30	25	9	2	1	0	0
Censored	0	1	4	18	25	26	27	27
Events	0	4	6	8	8	8	8	8
Q3								
At risk	35	30	22	11	3	2	2	1
Censored	0	3	9	19	24	25	25	25
Events	0	2	4	5	8	8	8	9
Q4								
At risk	35	23	16	9	7	5	0	0
Censored	0	5	6	13	15	17	21	21
Events	0	7	13	13	13	13	14	14

C

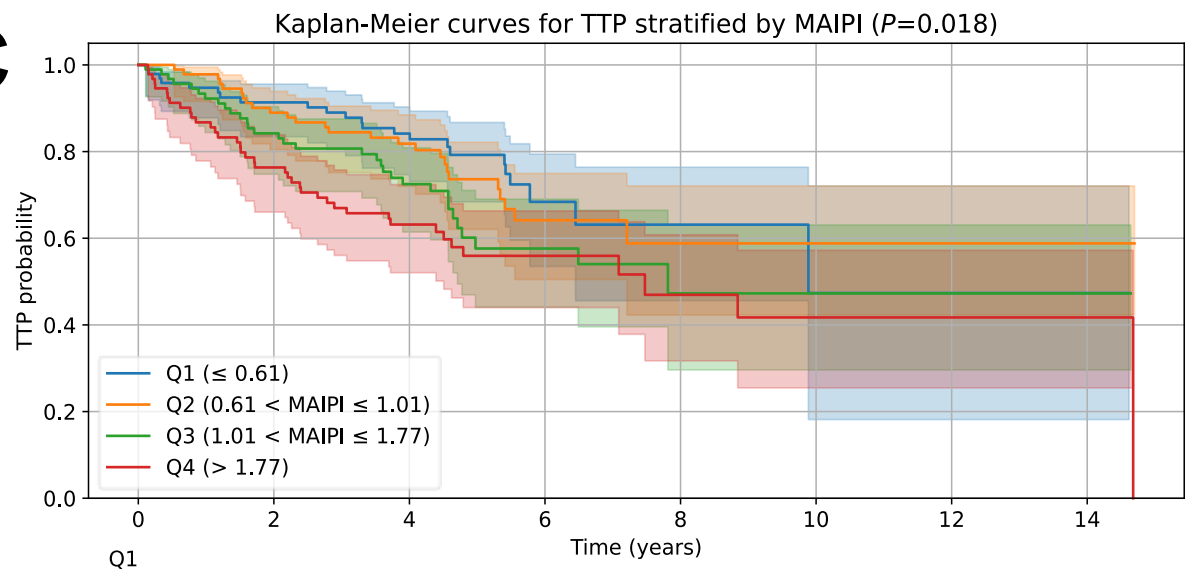

Q1								
At risk	98	81	64	16	4	3	2	1
Censored	0	9	20	61	72	72	73	74
Events	0	8	14	21	22	23	23	23
Q2								
At risk	97	80	57	21	7	6	2	1
Censored	0	7	24	51	64	65	69	70
Events	0	10	16	25	26	26	26	26
Q3								
At risk	97	72	50	17	6	6	4	1
Censored	0	11	24	50	59	59	61	64
Events	0	14	23	30	32	32	32	32
Q4								
At risk	96	66	42	15	10	7	5	1
Censored	1	10	23	46	49	51	53	57
Events	0	21	32	36	38	39	39	39

D

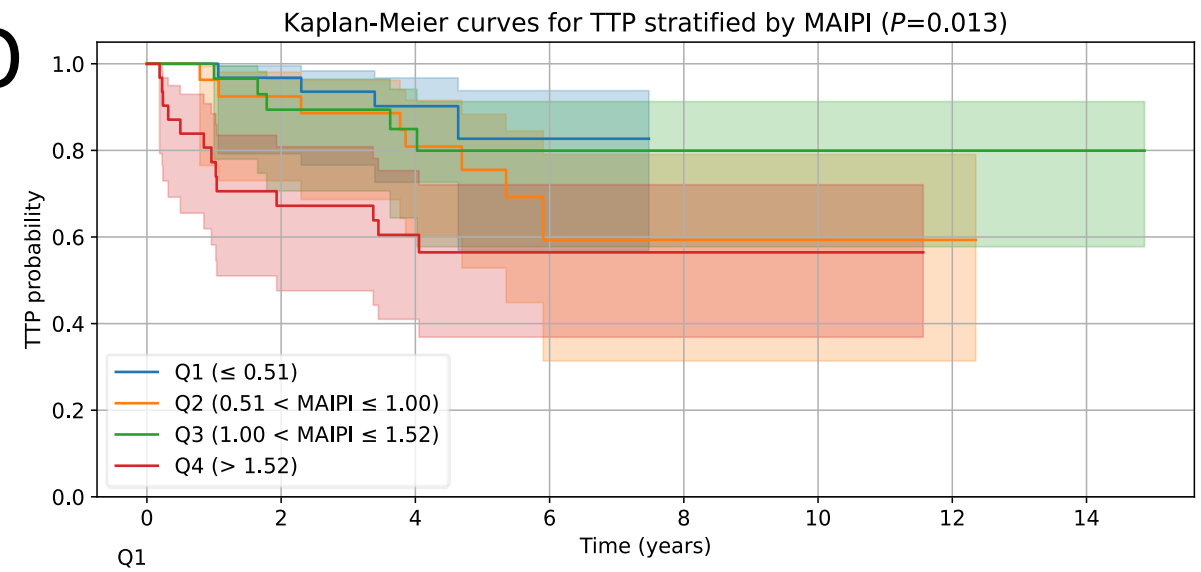

Q1								
At risk	32	30	20	5	0	0	0	0
Censored	0	1	9	23	28	28	28	28
Events	0	1	3	4	4	4	4	4
Q2								
At risk	31	24	18	6	2	2	1	0
Censored	0	5	8	17	21	21	22	23
Events	0	2	5	8	8	8	8	8
Q3								
At risk	31	24	18	5	2	2	1	1
Censored	0	4	9	21	24	24	25	25
Events	0	3	4	5	5	5	5	5
Q4								
At risk	32	20	15	2	1	1	0	0
Censored	0	2	5	17	18	18	19	19
Events	0	10	12	13	13	13	13	13
