# Supplementary material for: Mantle cell lymphoma artificial intelligence prognostic index using hematoxylin and eosin histology
Source: Leukemia. 2026 Jul 7;40(9):1961–7. doi: 10.1038/s41375-026-03036-8 (PMC13506322; doi:10.1038/s41375-026-03036-8)
Supplement: Supplementary file 3 [file 41375_2026_3036_MOESM4_ESM.pdf]

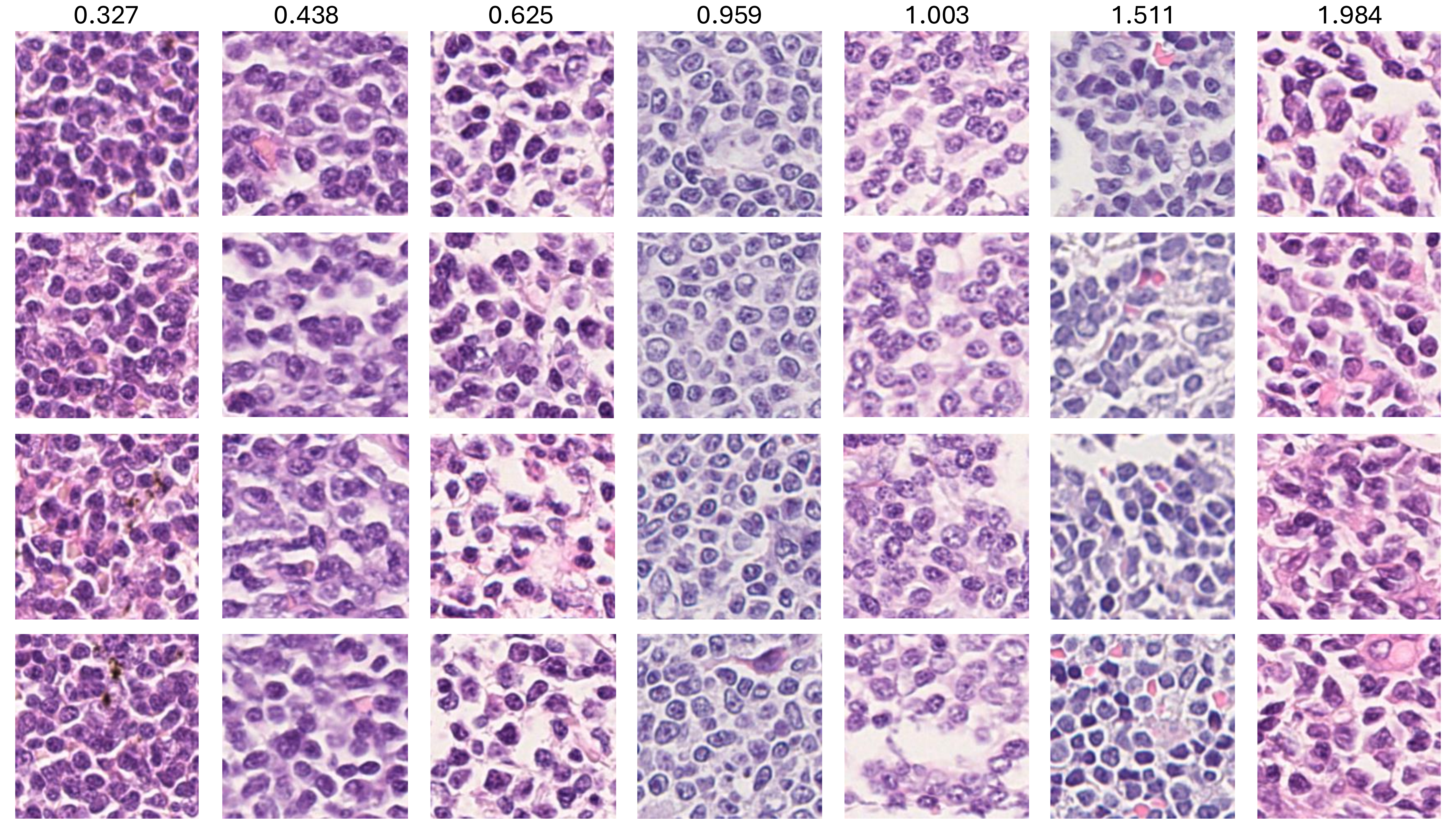
0.327
0.438
0.625
0.959
1.003
1.511
1.984
