# Supplementary material for: Mantle cell lymphoma artificial intelligence prognostic index using hematoxylin and eosin histology
Source: Leukemia. 2026 Jul 7;40(9):1961–7. doi: 10.1038/s41375-026-03036-8 (PMC13506322; doi:10.1038/s41375-026-03036-8)
Supplement: Supplementary file 4 [file 41375_2026_3036_MOESM1_ESM.docx]

**Supplementary**

**Supplementary Analysis**

We calculated the *C*-index of the clinical risk score MIPI for the development cohort, yielding values of *C=*0.664 (*P*<0.001) for OS and *C=*0.627 (*P*<0.001) for TTP. In the test cohort, the MIPI score achieves *C*-indices of *C=*0.654 (*P*=0.002) and *C=*0.703 (*P*<0.001) for OS and TTP, respectively. Since Ki67 is also significantly associated with OS and TTP in MCL and is part of the MIPIb score, we also calculated the *C*-index for MIPIb. The values achieved were *C=*0.739 (*P*<0.001) for OS and *C*=0.693 (*P*<0.001) for TTP in the development cohort, and *C=*0.686 (*P*=0.001) and *C=*0.732 (*P*<0.001) for OS and TTP in the test cohort, respectively (**Supplementary Table 2**).

**Supplementary Data**

**Supplementary Figure 1. Comparison of MIPI-, MIPIb-, and MAIPI-based risk stratification.** Kaplan-Meier curves for OS (A & C) and TTP (B & D) including all patients (n=568) using the MIPI (A & B) and MIPIb (C & D) combining the MIPI score and the Ki67 biomarker. Within p53 expression groups p53≤50% (E) and p53>50% (F) patients are stratified based on MAIPI for OS using the established threshold of 1.292. Within the high risk Ki67 group, the patients are further stratified by median MAIPI for OS (G) and TTP (H).

**Supplementary Figure 2. MAIPI quartiles stratify OS and TTP outcomes.** Kaplan-Meier curves for OS (A & B) and TTP (C & D) in the development cohort (A & C) and the independent test cohort (B & D). Patients were stratified by MAIPI quartiles, so each strata contains 25% of the patients from the respective cohort.

**Supplementary Figure 3. Example MCL patches with classic morphology.** Visualization of exemplary patches for seven patients with MAIPI for OS in ascending order. Four patches with classic morphology are shown for each patient to allow morphological inspection within this subgroup of MCL.

**Supplementary Table 1. Comparison of baseline characteristics between development and test cohorts.** A comparison of the population statistics between development and test cohort was performed. A *P*-value > 0.05 indicates no significant difference between the two cohorts. The mean and standard deviation (SD) are reported for the clinical parameters Ki67 and MIPI score. For the categorical MIPI group, the total counts and percentage of the total cohort are reported.

**Supplementary Table 2. Prognostic performance across scores.** For the two cohorts and the two times *C*-indices are reported for the clinical MIPI, the MIPIb and our established MAIPI and the two combined scores.

**Supplementary Table 3. Association between MAIPI and clinical outcomes assessed by Cox proportional hazards modeling.** OS and TTP were evaluated using the predicted Image Risk Score (MAIPI). Results are presented as hazard ratio coefficients (coef) with corresponding standard errors (se), 95% confidence intervals (CI), and *P*-values. Significance is indicated as *P* < 0.05.

**Supplementary Table 4. Multivariable Cox regression incorporating MAIPI and MIPI.** Models for OS and TTP include both the Image Risk Score (MAIPI) and MIPI score as covariates. Coefficients (coef), standard errors (se), 95% confidence intervals (CI), and *P*-values are shown for each variable, with statistically significant associations indicated as *P* < 0.05.

**Supplementary Table 5.** **Cox regression analysis of MAIPI, MIPI and Ki67 for outcomes.** Cox-regression analysis for OS and TTP. Hazard ratios expressed as coefficients (coef), standard errors (se), 95% confidence intervals (CI), and *P*-values are reported for each covariate including, Image Risk score (MAIPI), MIPI score and Ki67 biomarker. Statistically significant results are indicated by a *P*-value of less than 0.05.

**Supplementary Table 6.** **Cox regression models evaluating MAIPI alongside TP53 status.** Associations with OS and TTP are summarized for Image Risk Score (MAIPI) and TP53. Effect estimates are provided as coefficients (coef) with standard errors (se), 95% confidence intervals (CI), and *P*-values. Results reaching *P* < 0.05 are considered significant.
